# Factors affecting long-term myopic regression after corneal refractive surgery for civilian pilots in southwest China

**DOI:** 10.1186/s12886-024-03399-5

**Published:** 2024-04-02

**Authors:** Zhen Zhang, Lan xi Xiang, Ye Wu, Qi Li, Shan hua Ke, Long qian Liu

**Affiliations:** 1 Department of Ophthalmology, West China Hospital, Sichuan University, 37 Guoxue Xiang, Chengdu, Sichuan Province 610041 PR China; 2 Department of Ophthalmology, Chengdu Civil Aviation Medical Center, Chengdu, Sichuan Province PR China; 3 Department of Internal, Chengdu Civil Aviation Medical Center, Chengdu, Sichuan Province PR China

**Keywords:** Myopic regression, Corneal refractive surgery, Civilian pilot, Accommodative, Eye habit

## Abstract

**Background:**

The purpose of this study was to analyze myopic regression after corneal refractive surgery (CRS) in civilian pilots and to explore the factors that may cause long-term myopic regression.

**Methods:**

We included civilian pilots who had undergone CRS to correct their myopia and who had at least 5 years of follow-up. We collected retrospective data and completed eye examinations and a questionnaire to assess their eye habits.

**Results:**

A total of 236 eyes were evaluated in this study. 211 eyes had Intrastromal ablations (167 eyes had laser in situ keratomileusis, LASIK, 44 eyes had small incision lenticule extraction, SMILE) and 25 eyes had subepithelial ablations (15 eyes had laser epithelial keratomileusis, LASEK and 10 eyes had photorefractive keratectomy, PRK). The mean preoperative spherical equivalent (SE) was − 2.92 ± 1.11 D (range from − 1.00 to -5.00 D). A total of 56 eyes (23.6%) suffered from myopic regression after CRS. Comparisons of individual and eye characteristics between the regression and non-regression groups revealed statistically significant differences in age, cumulative flight time, postoperative SE (at 6 months and current), uncorrected visual acuity (UCVA), accommodative amplitude (AA), positive relative accommodation (PRA), postoperative period, types of CRS and eye habits. Generalized propensity score weighting (GPSW) was used to balance the distribution of covariates among different age levels, types of CRS, cumulative flying time, postoperative period and continuous near-work time. The results of GPS weighted logistic regression demonstrated that the associations between age and myopic regression, types of CRS and myopic regression, continuous near-work time and myopic regression were significant. Cumulative flying time and myopic regression, postoperative period and myopic regression were no significant. Specifically, the odds ratio (OR) for age was 1.151 *(P* = 0.022), and the OR for type of CRS was 2.769 (*P* < 0.001). The OR for continuous near-work time was 0.635 with a *P* value of 0.038.

**Conclusions:**

This is the first report to analyze myopic regression after CRS in civilian pilots. Our study found that for each year increase in age, the risk of civilian pilots experiencing myopic regression was increased. Intrastromal ablations had a lower risk of long-term myopia regression than subepithelial ablations. There is a higher risk of myopic progression with continuous near-work time > 45 min and poor accommodative function may be related factors in this specific population.

## Background

Considerable experience with corneal refractive surgery (CRS) has been gained worldwide. The aim is generally to allow the patient to do away with spectacles or contact lenses. Previously reported limitations of glasses and contact lenses such as displacement, reduced field of view and poor contrast sensitivity required for flight duty can all be overcome with CRS [1, 2]. The success rate is high, and complications following refractive surgery are infrequent, but refractive regression has been noticed and reported. The maintenance of visual acuity is important to the safe operation of an aircraft and the most important risks, from an aviation standpoint, are loss of best-corrected visual acuity, under correction or overcorrection, fluctuation in vision at different times of the day, glare, “halo” or “starburst” effects due to corneal haze, loss of contrast sensitivity, loss of low-contrast visual acuity, and regression toward preoperative refraction levels [3]. Pilots provide the particular challenge of requiring clear vision at a range of distances and viewing positions, including optimal distance vision for taxiing and approach [1]. For flight safety reasons, when the distance vision loss caused by myopic regression is not fit for the standards (one eye is < 0.7 Landolt rings), there are some visual acuity test requirements that apply to aeromedical assessment, civilian pilots need to wear spectacles or contact lenses again when they return to flying duties [4, 5]. CRS was used to correct refractive errors of a degree that previously prevented applicants from obtaining medical certification needed to work in the aviation environment in China before 2006 [6]. To increase the recruitment pool of potential civilian pilots, moderate myopia and CRS were approved for civilian pilots at initial examination from 2017 [7]. There is, however, rarely any reason for an applicant to submit to refractive surgery in order to meet the visual requirement. One of the most important risks civilian pilots might contemplate is regression towards pre-operative refraction levels and wear glasses again. Pilots are required to use glasses during the exercise of the privileges of the licence or rating applied for or held. But most of them won’t keep using glasses in their daily lives. Uncorrected refractive errors may cause accommodative and binocular dysfunctions and asthenopia. These potential adverse effects that could be incompatible with flying duties [3]. Due to the limitations of identification standards [4–7] and no enough cognition about CRS, the number of myopic civilian pilots choosing CRS is still relatively small, but long-term follow-up myopic regression has been noticed. Therefore, this matter should be investigated, as myopic regression and refractive errors often bring safety problems to civilian pilots.

Refractive regression after any CRS refers to the tendency of the human eye to shift toward its original refraction after a period of desired refraction has been achieved postoperatively [8]. The definition of regression has varied between studies and has traditionally been defined as residual myopia or hyperopia of 0.25dioptres (D) or greater change occurring during follow-up [9]. Specifically, refractive regression is caused by changes at the level of the cornea, not any refractive changes due to lenticular processes or changes in axial length. Because different definitions of regression, methods, and follow-up periods often prevent meaningful comparisons between studies, the exact incidence of refractive regression is difficult to ascertain. Any degree of myopia results in reduced visual acuity, and the correlation between the amount of myopic regression and visual acuity is not clear [10]. Vision chart of Landolt rings projectors is adopted as the test equipment in Civil Aviation Administration of China (CAAC). It is more difficult than other vision tests. For example, it should contain ten symbols in each row with 8 random gaps, no error should be allowed per line of ten symbols. Examiners should not allow the applicant to squint during testing as using the eyelids as a stenopaeic slit may mask refractive errors. In many pilots there is a reluctance to wear spectacles so they may have “overachieved” on the subjective vision test. Aeromedical examiner (AME) found that a significant degree of myopia, i.e. -0.50D or more, will be detected difficult in finishing all symbols of 0.7 (Landolt rings) during the screening examination, provided the applicants is not allowed to squint. Therefore, we defined the regression group as pilots having myopia of more than 0.50D and uncorrected distant vision lower than 0.7 following CRS procedures based on the spherical equivalent (SE) of noncycloplegic refraction and uncorrected distant vision in our study. The definition criteria are less stringent than in other studies but can reflect the visual performance and needs of the airman more comprehensively from an aeromedical significance standpoint.

To date, the exact mechanism of refractive regression after CRS has not been elucidated. Many factors are postulated to be involved. Most observations are mainly related to corneal epithelial thickening and corneal biomechanical changes. The main risk factors are preoperative refractive [8], corneal thickness and the amount of correction [11], age [9], dry eye [12], surgical method [13], etc. Surgical method can be divided in two categories: Intrastromal ablations and subepithelial ablations. Different types and methods of CRS have the problem of refractive regression [14]. Currently, high myopia is considered a risk factor for myopic regression following laser refractive surgery. Our previous study has evaluated the safety of CRS in civilian pilots in China [15], however, the regression following CRS among this population has not been reported. So far, there is limited research on postoperative outcomes for moderate to low myopia and even fewer studies on the long-term myopic regression of patients without postoperative problems.

The purpose of this study was to analyze the myopic regression rate of civilian pilots after CRS for at least 5 years in southwest China and to explore the risk factors that may cause long-term myopia regression.

## Methods

### Subjects

This study is part of a large study to evaluate the safety of CRS in civilian pilots in China, aiming to analyze the long-term myopia regression rate after CRS and explore the risk factors causing myopia regression in civilian pilots. The study protocol was approved by the hospital’s institutional review board [No 2014(33), 1-6-2015] and conducted according to the Declaration of Helsinki. Written informed consent was obtained from all participants.

Civilian pilots who have had CRS and are being considered for medical certification or recertification should meet the following criteria in China [5, 7]: (a) The surgery is performing surface or flap corneal refractive surgery using an excimer laser or a femtosecond laser. (b) Vision is stable. (c) There is no corneal haze and no complaints of glare, halos or “ghosting”. (d) The result meets the visual requirements, and the assessment must be based on measurements made by a qualified vision care specialist acceptable to the Licensing Authority. (e) There should be follow-up examinations by a qualified vision care specialist six months after return to duty and yearly thereafter. The enrolled participants were grouped into a myopic regression group (uncorrected distant vision lower than 0.7 and with a degree of myopia of -0.5 D or more) and a non-regression group (uncorrected distant vision 0.7 or better and with a degree of myopia less than 0.5 D) based on the SE of noncycloplegic refraction and uncorrected distant vision. For those who applied to only one of the criteria were grouped by uncorrected distant vision only, but we don’t have this population in our sample.

The inclusion criteria were as follows: (1) aged 18–35 years; (2) unremarkable general and ocular health; (3) best-corrected visual acuity of at least 1.0 Landolt rings; (4) no myopic regression within 5 years after surgery: and (5) surgery including laser in situ keratomileusis, LASIK, small incision lenticule extraction, SMILE, photorefractive keratectomy, PRK, laser epithelial keratomileusis, and LASEK. The exclusion criteria included ocular pathology, retinal disorders, glaucoma and postoperative period of less than 5 years. We excluded those whose vision is not stable, those who had corneal haze or complications of glare, halos or “ghosting” and those who did not finish the follow-up examinations by a qualified vision care specialist after return to duty.

The two groups were asked to cooperate as retrospective data was collected, were asked to complete a questionnaire to assess their eye habits, and were requested to complete ocular examinations.

The procedures included the following:

### Data collection and eye habits questionnaire

Retrospective data were collected from the treating hospital, including preoperative SE (D), age at surgery, types of surgery, and postoperative review records. 8 experienced surgeons performed all of the procedures. All pilots were examined by ophthalmologic tests preoperatively and at 6 months and every year after surgery by AME. 132 civilian pilots completed the eye habits questionnaire before the periodic ophthalmologic re-examinations by the AME which was conducted between October 2020 and April 2021. The collected data included gender, age, cumulative flying time, weekly outdoor activity time, daily screen time and continuous near-work time. Near-work includes activities done at a short working distance, such as reading books or writing, mobile phone or computer use/ playing video games, or watching TV /video, etc. [16, 17].

### Ocular examinations

As an internationally uniform standard, we adopted Landolt rings as the distance vision test symbol, which is also needed to be used for civil aviation pilots in China. Uncorrected visual acuity (UCVA), best spectacle corrected visual acuity (BSCVA), manifest refraction, slit lamp and fundus examination, intraocular pressure (IOP), sodium fluorescein staining and corneal topography were examined [18]. The refractive examination was performed using static retinoscopy and subjective refraction (RT-600, Nidek Co. Ltd.). According to China’s 2013 expert consensus for the diagnosis and treatment of dry eyes, any one of the symptoms of dry eyes, including burning sensation and foreign body sensation, accompanied by a break-up time (BUT) ≤ 5 s, can be diagnosed as dry eye [19]. The IOP examination was performed using a CT-80 tonometer (Topcon, Japan).

Accommodative tests: accommodative amplitude (AA)was measured by the push-up method with a single 20/30 Snellen line target in free space. Positive and negative relative accommodation(PRA and NRA); were measured with plus and minus lenses, respectively, using an accommodative target of 20/30 visual acuity at 40 cm until a sustained blur was perceived. Monocular accommodative facility (MAF); was measured by the same method but without polarized glasses and with the nonviewing eye occluded. Binocular accommodative facility (BAF); was measured at 40 cm using ± 2.00 D flipper lenses and the 20/30 letter line on the Vectogram9 (Tianjin O put Visual Training Co. Ltd.), which included suppression control for the binocular measurement. Dynamic retinoscopy with the monocular estimate method at 40 cm was performed with the result of the subjective refraction placed in a trial frame while using trial lenses.

### Data analysis

Statistical software R (Version 4.1.1; R Core Team, 2021) was used for data analysis and description. Descriptive statistics such as absolute and relative frequencies for discrete parameters as well as the mean and standard deviation for continuous parameters were computed. Independent sample t-test and ${\text{x}}^{2}$ test were used to compare characteristics of included participants between the regression and non-regression groups. Generalized propensity score weighting (GPSW) was used to balance covariates that both related with exposure and outcome variables. Then GPS weighted logistic regression was applied to detect related factors and outcome variables. The significant level was set as α = 0.05. A *P* value less than and equal to 0.05 was considered statistically significant.

## Results

### Demographic data

A total of 132 civilian pilots had undergone CRS in Southwest China; 2 did not satisfy the inclusion criteria given that they were older than 35; 10 were excluded for a postoperative period of less than 5 years; and 1 was diagnosed with glaucoma. After these exclusions, 236 eyes were evaluated in this study (2 of 119 pilots had one eye CRS). A total of 211 eyes had Intrastromal ablations (167 eyes had LASIK, 44 eyes had SMILE) and 25 eyes had subepithelial ablations (15 eyes had LASEK and 10 eyes had PRK). The mean preoperative SE was − 2.92 ± 1.11 D (range from − 1.00 to −5.00 D). A total of 56 eyes (23.6%) suffered from myopic regression after CRS. The pilot’s preoperative data are shown in Table 1. Age at surgery and preoperative SE were similar in the two groups.

### Comparisons of individual and eye characteristics in regression and non-regression groups

When comparing individual and eye characteristics, there were statistically significant differences in age, cumulative flight time, types of CRS, postoperative SE (at 6 months and current), UCVA, AA and PRA between the regression and non-regression groups (Table 1). Differences in the postoperative period (which means 5–16 years postoperatively), types of CRS and eye habits were statistically significant (Table 2).

**Table 1:** Demographic, percentages and clinical findings of the subjects in both groups

	Regression group (*N* = 56)	Non-regression (*N* = 180)	$\varvec{t}/{\varvec{x}}^{2}/\varvec{Z}$	P-value
Gender (M/F)	30/0	88/3		1.000
Age (years)	32.03 ± 3.78	29.18 ± 3.32	3.895	< 0.001
Age at surgery (years)	20.00 (19.00, 24.00)	20.00 (18.00, 22.00)	0.863	0.388
Cumulative flying time (hours)	5,000.00 (2,800.00, 8,200.00)	3,200.00 (1,762.50, 5,500.00)	2.323	0.020
Types of CRS			21.639	< 0.001
LASIK	32(19.2)	135(80.8)		
SMILE	10(22.7)	34(77.3)		
LASEK	6(40.0)	9(60.0)		
PRK	8(80.0)	2(20.0)		
Preoperative SE(D)	−3.12 ± 1.16	−2.86 ± 1.09	−1.541	0.125
Postoperative data (at 6 months)				
SE(D)	+ 0.80 ± 0.22	0.96 ± 0.26	−4.1706	0.000
CCT	471.48 ± 27.39	478.90 ± 42.09	−1.2393	0.216
Postoperative SE	−1.11 ± 0.85	0.15 ± 0.44	−10.694	<0.001
UCVA	0.48 ± 0.19	0.98 ± 0.08	−19.373	<0.001
IOP (mmHg)	12.76 ± 3.00	12.60 ± 2.85	0.346	0.729
Accommodative test				
AA (right eye only,D)	9.07 ± 1.96	10.02 ± 1.94	−3.186	0.002
BAA	8.91 ± 2.00	10.00 ± 1.97	−2.566	0.012
BAF (cpm)	13.00 (8.75, 17.00)	13.00 (9.00, 16.00)	0.079	0.937
MAF (right eye only,D)	13.00 (8.00, 16.00)	12.50 (8.00, 15.00)	0.254	0.799
NRA(D)	1.92 ± 0.71	1.90 ± 0.56	0.187	0.851
PRA(D)	1.83 ± 1.82	2.76 ± 1.78	−2.423	0.017
MEM (right eye only,D)	0.03 ± 0.37	−0.09 ± 0.52	1.517	0.130
MEM	0.04 ± 0.33	−0.11 ± 0.42	1.847	0.067

CRS Corneal refractive surgery, LASIK Laser in situ keratomileusis, SMILE Small incision lenticule extraction, PRK Photorefractive keratectomy, LASEK Laser epithelial keratomileusis, SE Spherical equivalent, UCVA uncorrected visual acuity, IOP Intraocular pressure. AA Accommodative amplitude, BAA Binocular Accommodative amplitude, BAF Binocular accommodative facility, MAF Monocular accommodative facility, MEM Monocular estimated method, PRA Positive relative accommodation, NRA Negative relative accommodation

**Table 2:** Demographics, percentages and clinical findings of the subjects in both groups

	Regression group (*N* = 56)	Non-regression (*N* = 180)	$\varvec{t}/{\varvec{x}}^{2}/\varvec{Z}$	P-value
Age at surgery, n(%)			<0.001	0.975
<20 ≥ 20	24(42.9) 32(57.1)	78(43.3) 102(56.7)		
Postoperative period (years), n(%);			17.450	< 0.001
5–10	26 (46.4)	135 (75.0)		
11–16	30 (53.6)	45 (25)		
Types of CRS, n(%)			16.090	< 0.001
Intrastromal ablations	42 (75.0)	169 (93.9)		
subepithelial ablations	14 (25.0)	11 (6.1)		
Preoperative SE(D), n(%);			1.723	0.189
−0.5D～−3.0D	23(41.1)	92(51.1)		
−3.0D～−5.0D	33(58.9)	88(48.9)		
Dry eye test, n(%)			0.025	0.873
Non-dry eye	31 (55.4)	98 (54.4)		
Dry eye	25 (44.6)	82 (45.6)		
Eye habits				
Weekly outdoor activity time, n(%)			25.823	< 0.001
≤ 5 h	18(62.1)	13(14.4)		
>5 h	11(37.9)	77(85.6)		
Daily screen time, n(%)			18.631	< 0.001
≤5 h	4(13.8)	20(22.2)		
5 to 8 h	16(55.2)	67(74.4)		
>8 h	9(31.0)	3(3.4)		
Continuous near-work time, n(%)			13.910	< 0.001
>45 min	18 (62.1)	22 (24.4)		
≤ 45 min	11(37.9)	68(75.6)		

CRS Corneal refractive surgery, SE Spherical equivalent

### Factors influencing refractive regression - based on generalized propensity score weighting (GPSW)

#### Age

**Fig. 1:**
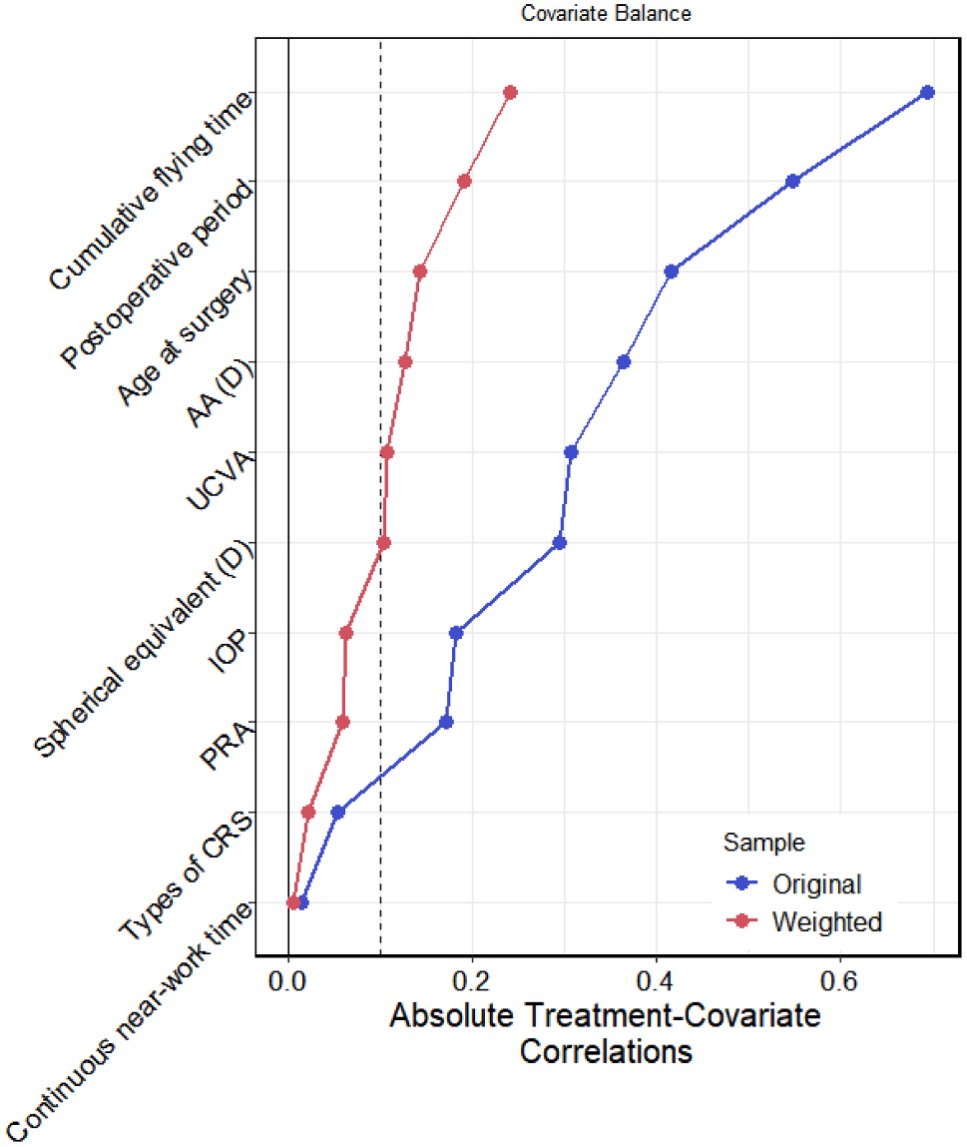
Correlation coefficients between age and covariates before and after weighting with propensity scores

Generalized propensity score weighting (GPSW) was used to balance the distribution of covariates among different age groups. After weighting, the mean absolute value of the correlation coefficient between age and covariates was 0.1 (≤ 0.1), indicating that the covariates were balanced, as shown in Table 3 and Fig. 1. The results of GPS weighted logistic regression demonstrated that the association between age and myopic regression was significant. Specifically, the odds ratio (OR) for age was 1.151 (*P* = 0.022), indicating that the OR for myopic regression was 1.151 with every one-year increase, Table 4.

**Table 3:** Correlation coefficients between age and covariates before and after weighting with propensity scores– age

Variables	Type	Uncorrected	Corrected	Status
Cumulative flying time	Binary	−0.693	−0.242	balanced
Continuous near-work time	Binary	−0.015	−0.005	balanced
AA	Continuous	−0.363	−0.127	balanced
PRA	Continuous	−0.170	−0.059	balanced
Types of CRS	Binary	0.054	0.022	balanced
Postoperative period	Binary	0.547	0.190	balanced
Spherical equivalent	Continuous	−0.294	−0.104	balanced
UCVA	Continuous	−0.306	−0.108	balanced
Age at surgery	Continuous	0.415	0.143	balanced
IOP	Binary	0.181	0.063	balanced

**Table 4:** Logistic regression model of the association between age weighted with propensity scores and myopic Regression

Variables	B	SE	Z	P value	OR	LCI	HCI
Intercept	−5.474	1.864	−2.936	0.003	0.004	< 0.001	0.162
Age	0.141	0.061	2.296	0.022	1.151	1.021	1.298

#### *Types of CRS*

**Fig. 2:**
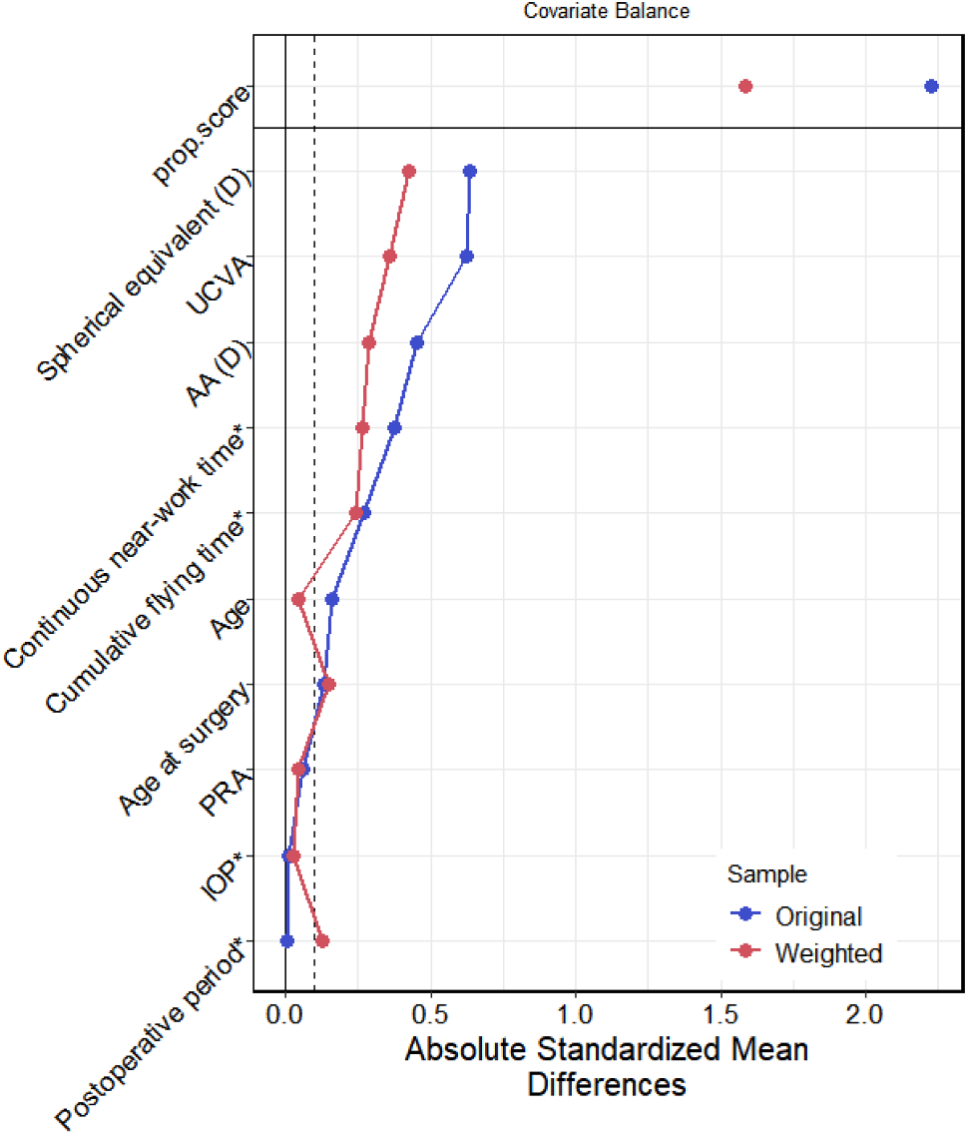
Correlation coefficients between types of surgery and covariates before and after weighting with propensity scores

Propensity score weighting (PSW) was used to balance the distribution of covariates between the intrastromal ablations and subepithelial ablations groups. The mean absolute difference after PSW was 0.196 (> 0.1), indicating that the covariates were not balanced (Table 5; Fig. 2). Therefore, the conclusion based on the following propensity score weighted logistic regression might still be affected by confounding effects. The results of the PS weighted logistic regression demonstrated that the association between the types of CRS with myopic regression was significant, where the OR for types of CRS = 2.769 (*P* < 0.001). This indicated that the risk of refractive regression in the subepithelial ablations group was 2.769 times that in the intrastromal ablations group (Table 6).

**Table 5:** Correlation coefficients between types of surgery and covariates before and after weighting with propensity scores– types of surgery

Variables	Type	Uncorrected	Corrected	Status
Age	Continuous	0.161	−0.043	balanced
Cumulative flying time	Binary	−0.270	−0.240	balanced
Continuous near-work time	Binary	−0.372	−0.266	unbalanced
AA	Continuous	−0.453	−0.285	unbalanced
PRA	Continuous	−0.061	−0.045	balanced
Postoperative period	Binary	0.002	−0.127	balanced
Spherical equivalent	Continuous	−0.632	−0.425	unbalanced
UCVA	Continuous	−0.623	−0.360	unbalanced
Age at surgery	Continuous	−0.129	−0.150	balanced
IOP	Binary	0.010	0.025	balanced

**Table 6:** Logistic regression model of the association between type of surgery weighted with propensity scores and myopic regression

Variables	B	SE	Z	*P* value	OR	LCI	HCI
Intercept	−2.344	0.372	−6.304	< 0.001	0.096	0.046	0.199
Types of CRS	1.019	0.246	4.147	< 0.001	2.769	1.711	4.481

#### *Continuous near-work time*

**Fig. 3:**
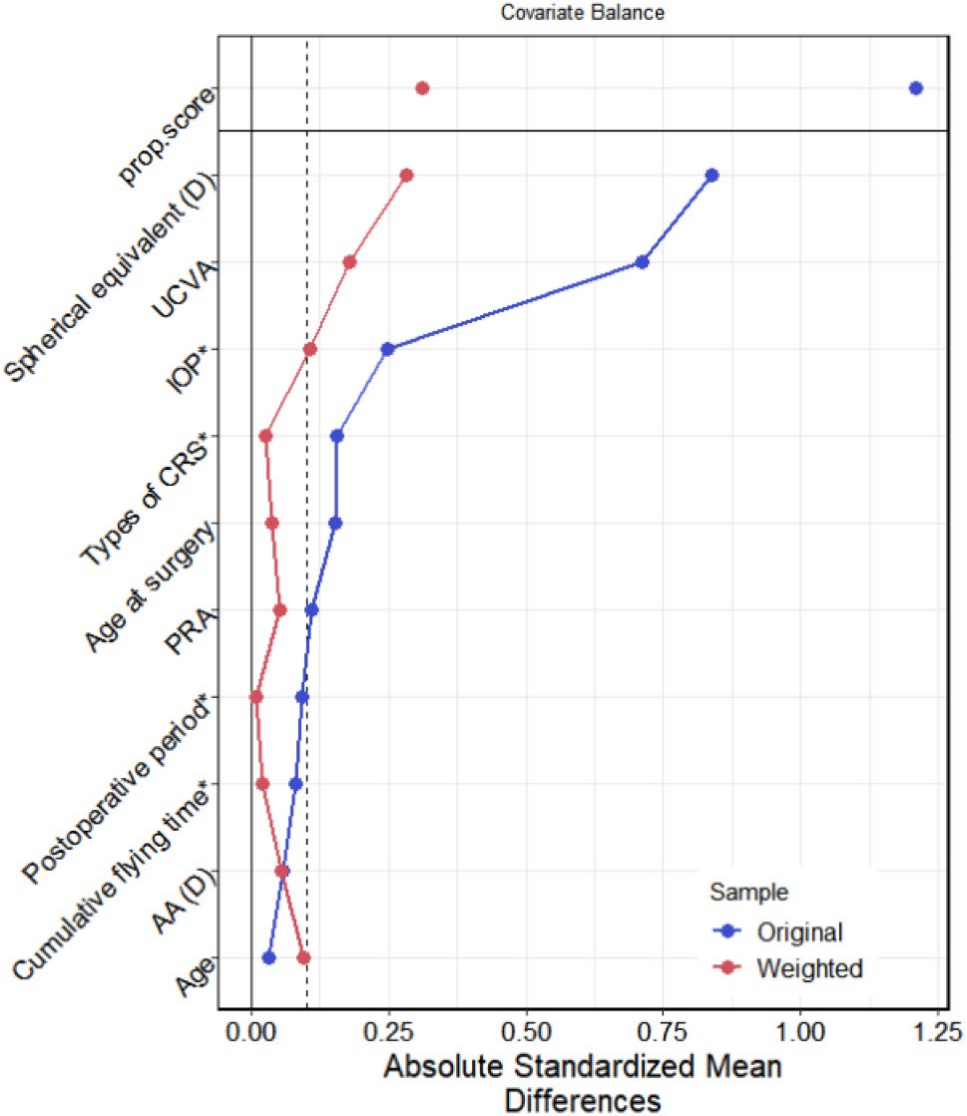
Correlation coefficients between eye habits and covariates before and after weighting with propensity scores– continuous near-work time

PSW was used to balance the covariates between different times of continuous near-work. The weighted standardized mean difference of the absolute values between groups is 0.086 (≤ 0.1), indicating that the covariate levels were balanced. Please refer to Table 7; Fig. 3 for more details.

The analysis results of the logistic regression model after propensity score weighting show that there is a statistically significant association between continuous near-work time and myopic progression. Specifically, the OR for continuous near-work time was 0.635, with a *p* value of 0.038, indicating that there was a higher risk of myopic progression in the pilots with continuous near-work time > 45 min than in the pilots with continuous near-work time < 45 min. Please see Table 8 for more information.

**Table 7:** Correlation coefficients between eye habits and covariates before and after weighting with propensity scores– continuous near-work time

Variables	Type	Uncorrected	Corrected	Status
Age	Continuous	−0.033	0.094	balanced
Cumulative flying time	Binary	0.081	−0.021	balanced
AA	Continuous	−0.059	−0.053	balanced
PRA	Continuous	0.110	0.051	balanced
Types of CRS	Binary	−0.155	−0.027	balanced
Postoperative period	Binary	−0.092	0.009	balanced
Spherical equivalent	Continuous	0.838	0.281	unbalanced
UCVA	Continuous	0.712	0.177	balanced
Age at surgery	Continuous	0.153	0.038	balanced
IOP	Binary	0.247	0.106	balanced

**Table 8:** Logistic regression model of the association between eye habits weighted with propensity scores and myopic regression

Variables	B	SE	Z	P value	OR	LCI	HCI
Intercept	−0.825	0.15	−5.509	< 0.001	0.438	0.327	0.588
Continuous near-work time	−0.454	0.219	−2.070	0.038	0.635	0.413	0.976

#### *Cumulative flying time*

**Fig. 4:**
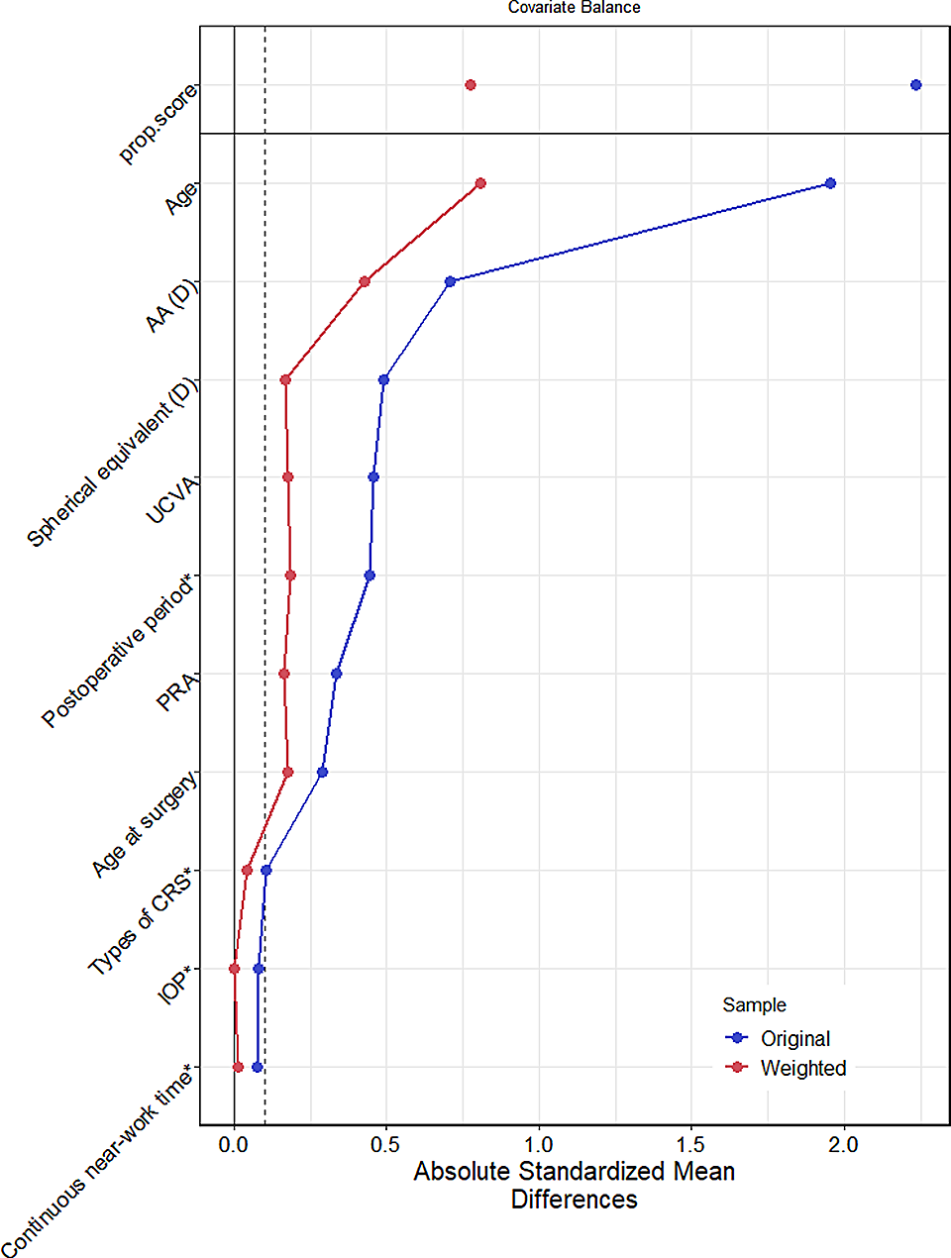
Correlation coefficients between cumulative flying time and covariates before and after weighting with propensity scores

GPSW was used to balance the distribution of covariates among different cumulative flying time. After weighting, the mean absolute value of the correlation coefficient between cumulative flying time and covariates was 0.21 (>0.1), indicating that the covariates were unbalanced, as shown in Table 9 and Fig. 4. Therefore, the conclusion based on the following propensity score weighted logistic regression might still be affected by confounding effects. The results of GPS weighted logistic regression demonstrated that the association between cumulative flying time and myopic regression was no significant (OR = 0.649, *P* = 0.073) (Table 10).

**Table 9:** Correlation coefficients between cumulative flying time and covariates before and after weighting with propensity scores– Cumulative flying time

Variables	Type	Uncorrected	Corrected	Status
Age	Continuous	−1.955	−0.807	unbalanced
Continuous near-work time	Binary	0.077	−0.014	balanced
AA (D)	Continuous	0.706	0.426	unbalanced
PRA	Continuous	0.334	0.164	balanced
Types of CRS	Binary	−0.107	−0.041	balanced
Postoperative period	Binary	−0.444	−0.185	balanced
Spherical equivalent (D)	Continuous	0.490	0.168	balanced
UCVA	Continuous	0.457	0.178	balanced
Age at surgery	Continuous	−0.290	−0.176	balanced
IOP	Binary	−0.080	0.002	balanced

**Table 10:** Logistic regression model of the association between cumulative flying time weighted with propensity scores and myopic regression

Variables	B	SE	Z	P value	OR	LCI	HCI
Intercept	−0.984	0.165	−5.97	< 0.001	0.374	0.271	0.516
Cumulative flying time	−0.432	0.241	−1.792	0.073	0.649	0.405	1.041

### Postoperative period

**Fig. 5:**
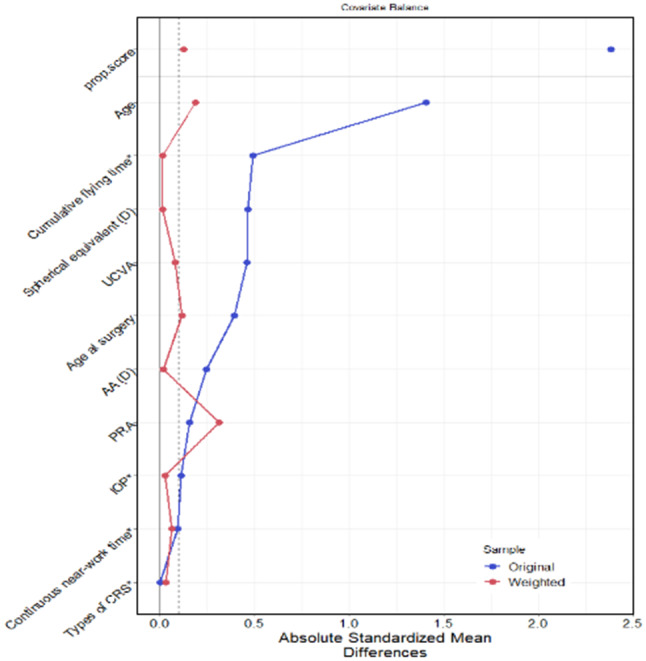
Correlation coefficients between postoperative period and covariates before and after weighting with propensity scores

PSW was used to balance the distribution of covariates between the postoperative period 5–10 years and 11–16 years groups. The mean absolute difference after PSW was 0.087 (<0.1), indicating that the covariates were balanced (Table 11; Fig. 5). The results of the PS weighted logistic regression demonstrated that the association between the postoperative period with myopic regression was no significant, where the OR for postoperative period = 1.117 (*P* = 0.611) (Table 12).

**Table 11:** Correlation coefficients between postoperative period and covariates before and after weighting with propensity scores– Postoperative period

Variables	Type	Uncorrected	Corrected	Status
Age	Continuous	1.405	0.186	balanced
Cumulative flying time	Binary	−0.491	0.013	balanced
Continuous near-work time	Binary	−0.097	0.065	balanced
AA (D)	Continuous	−0.244	−0.017	balanced
PRA	Continuous	−0.157	0.312	unbalanced
Types of CRS	Binary	0.001	−0.033	balanced
Spherical equivalent (D)	Continuous	−0.467	−0.016	balanced
UCVA	Continuous	−0.461	0.082	balanced
Age at surgery	Continuous	−0.392	−0.118	balanced
IOP	Binary	0.111	0.028	balanced

**Table 12:** Logistic regression model of the association between postoperative period weighted with propensity scores and myopic regression

Variables	B	SE	Z	P value	OR	LCI	HCI
Intercept	−1.283	0.344	−3.725	< 0.001	0.277	0.141	0.544
Postoperative period	0.111	0.218	0.508	0.611	1.117	0.729	1.713

## Discussion

Research has found that older patients with high myopia are more prone to experience myopic regression than younger patients, suggesting a correlation with the surgeon’s tendency toward under-correction to compensate for the progressive loss of accommodation by age [20, 21].The age range for pilots opting for CRS is usually concentrated between high school graduation and college, so the time of surgery among the pilots in our study is relatively centered at 18 to 23, with an average age of 20. Analysis of postoperative data (at 6 months) indicates that surgeons choose slight overcorrection during the surgical design phase to prevent myopic regression, hence, no cases of myopic regression were observed in the early postoperative period. However, there was statistically significant differences in postoperative period (5–16 years) and cumulative flight time between the two groups. Civilian pilots start their cumulative flying time in their third year of university, around at age of 20, studying flight practical training. Further comparison was made divided by age and no statistically significant difference was found in terms of myopic regression. This indicates that choosing CRS around at age of 20 might not have a general impact on myopic regression. GPSW was used in our data analysis and the results of the PS weighted logistic regression demonstrated that the association between postoperative period with myopic regression and cumulative flight time with myopic regression was no significant (*P* = 0.611 and *P* = 0.073). This indicates that different postoperative period and cumulative flight time might not have a general impact on myopic regression in pilots. Current research suggests that cellular proliferation events play a significant role in myopic regression [13]. It has been noted that epithelial compensation and corneal stroma undergoing longitudinal morphological changes can lead to refractive regression over time [22, 23]. Our study found that for each year increase in age, the risk of pilots experiencing refractive regression increased by a factor of 1.151. A study by Lim et al. [18] also found progressive myopic regression and corneal thickening in moderate myopic eyes during a 10-year follow-up after CRS, which is consistent with our results. After ruling out noncorneal factors, the reason for myopic regression in pilots, related to age, is more inclined toward biomechanical changes in the cornea after corneal ablation or environmental risk factors. Further studies are needed in this area.

CRS have potential adverse effects that could be incompatible with flying duties, including corneal scarring or opacities, worsening or variability of vision, and night glare [3]. Only 22 pilots in the Chinese Air Force chose CRS by 2023, all of which were LASEK or PRK, intrastromal ablations have not been implemented [2]. The advantages of subepithelial ablations include no residual corneal flap, thick postoperative corneal stromal thickness, and fewer surgically induced higher-order aberrations. The proportion of choosing CRS is only 5.50% in civilian pilots with refractive errors in southwest China [24]. We found that the long-term myopic regression rates for pilots who chose intrastromal ablations group and subepithelial ablations group were 19.9% and 56%, respectively. The results of the propensity score (PS)-weighted logistic regression demonstrated that the association between the two groups with myopic regression was significant, which indicated that the risk of refractive regression in the subepithelial ablations group was 2.769 times that in the intrastromal ablations group. However, the conclusion based on the following propensity score weighted logistic regression might still be affected by confounding effects for the mean absolute difference after PSW was 0.196 (> 0.1). Further comparison was made divided by types of surgery and we found that the long-term myopic regression rates for pilots who chose LASIK, SMILE, LASEK, PRK were 19.2%, 22.7%, 40% and 80%, respectively. However, the small number of participants in the subepithelial ablations group was a limitation. Song et al. [2] found that subepithelial ablations can significantly improve the visual acuity and refractive error of military pilots in the early stage, and ensure effectiveness, stability and safety. However, a follow-up is needed to understand the natural course of LASEK and PRK. Lim et al. [18] found that the myopic regression rates after 10 years of LASIK and LASEK for moderate myopia were 66.7% and 73.0%, respectively, with average myopia changes of −1.09 D and − 1.34 D, respectively, indicating progressive myopic regression. Chen et al. [25] suggested that the myopic regression rate for LASIK is approximately 21% (5.5–22.7%). Naderi et al. [26] estimated that the myopic regression rate after PRK is approximately 19%. Different research results may be attributed to variations in study subjects and observation periods. Kuryan et al. [27] in a randomized controlled trial, found uncertainty regarding better refractive and visual outcomes between LASEK and LASIK in patients with low to moderate myopia.

Myopia results from complex genetic and environmental causes. Environmental risk factors that have been determined to be associated with myopia include continuous near-work time and little outdoor exposure [16, 28]. Previous studies have shown that increased near-work time can cause asthenopia, affect myopia development [29], and increase the incidence of myopia [16]. In our study, there was a statistically significant association between eye habits and myopic progression. Specifically, the OR for continuous near-work time is 0.635 with a *p* value of 0.038, indicating that there is a higher risk of myopic progression in the pilots with continuous near-work time > 45 min. Several studies in adults have demonstrated gene‒environment interactions for refractive error, particularly with accumulated near-work activity [30–32]. Since myopia genes are common in the population, adjustment of lifestyle should be a major focus in the prevention of myopia. For myopic patients, consciously limiting the time of continuous near work can not only delay the progression of myopia but also reduce asthenopia [17]. Myopic pilots receiving CRS should be aware of the prevention of myopia regression by lifestyle factors.

Accommodative dysfunctions (ADs) are significant potential risk factors for the progression of myopia [33], so we analyzed accommodative parameters, including AA, PRA/NRA, AF, and MEM, in this study. There were statistically significant differences in AA and PRA between the two groups. The accommodative function in the myopic regression group was poorer. This may be attributed to the fact that pilots in the postoperative period experienced myopic regression but did not consistently wear glasses. It is also possible that eyes with lower amplitudes of accommodation must use more of their accommodative reserve for near work. Myopia may be an adaptation that develops in eyes with reduced accommodative amplitudes [34]. Many findings may explain that the poor performance and limited accommodative function are associated with age [35, 36]. The result is consistent with the progressive loss of accommodation by age in this study, though our study did not have data for people over 35 years old. The relationship between accommodation and myopia has long been a subject of interest, as myopia usually accompanies ADs [37]. From the current research, most studies on postoperative accommodative function after CRS have been positive [38], but there is no literature on the study of accommodative function in postoperative myopic regression. While the causal relationship between accommodation and myopia is still debated, the involvement of accommodative function in the emmetropization process of the eye is widely accepted. Therefore, we speculate that if we can improve the accommodative function of postoperative myopic regression pilots by visual training [39], it may effectively alleviate or even prevent further regression.

CRS is a cause of iatrogenic dry eye syndrome [40], and chronic dry eye may lead to a higher regression rate in CRS patients after surgery [40, 41] Treatment with drugs to lower intraocular tension can effectively reduce the incidence of corneal ectasia and early myopic regression [42]. In this study, the 236 eyes included had a preoperative refraction of less than − 5.0 D, with an average of −2.92 ± 1.11 D and an average intraocular pressure of 12.74 ± 2.97 mmHg. Myopic regression occurred in 23.1% of pilots with dry eyes and 24% of pilots with non-dry eyes(*p*>0.05). Negative results may be related to the selection of the subjects, as civilian pilots have strict aeromedical restrictions when they choose CRS and resume airman duties [5]. Overall, impotent observations that would help us in the daily clinic.

To date, factors affecting myopic regression after CRS for civilian pilots have never been reported. There is little knowledge about the long-term myopia regression of patients with low to moderate myopia after surgery. There are some limitations in this study. The number of participants in our study was small and observation in different follow-up years is needed to understand the course of myopic regression after CRS. We did not check current epithelial maps. The surgical technique was not effectively controlled because the surgeon that performed the surgery and the equipment used were not the same between groups. A total of 75 eyes (31.8%) after CRS more than 10 years. These data were based on the currently outdated broad beam laser rather than contemporary techniques, such as femtosecond laser technology. Nevertheless, preoperative and early postoperative data were well controlled between the regression and non-regression pilots. The interference caused by early postoperative myopia regression was eliminated.

## Conclusions

Our study found that significant differences in age, cumulative flight time, postoperative SE (at 6 months and current), UCVA, AA, PRA, postoperative period, types of CRS and eye habits between the regression and non-regression groups. For each year increase in age, the risk of civilian pilots experiencing myopic regression was increased. Intrastromal ablations had a lower risk of long-term myopia regression than subepithelial ablations. There is a higher risk of myopic progression with continuous near-work time > 45 min and poor accommodative function may be related factors in this specific population.

## Data Availability

No datasets were generated or analysed during the current study.

## Abbreviations

CRS: Corneal refractive surgery
LASIK: Laser in situ keratomileusis
SMILE: Small incision lenticule extraction
PRK: Photorefractive keratectomy
LASEK: Laser epithelial keratomileusis
SE: Spherical equivalent
UCVA: Uncorrected visual acuity
IOP: Intraocular pressure
AA: Accommodative amplitude
BAF: Binocular accommodative facility
MAF: Monocular accommodative facility
MEM: Monocular estimated method
PRA: Positive relative accommodation
NRA: Negative relative accommodation
ADs: Accommodative dysfunctions
GPSW: Generalized propensity score weighting
